# Shifting between self-governing and being governed: a qualitative study of older persons’ self-determination

**DOI:** 10.1186/1471-2318-14-126

**Published:** 2014-11-28

**Authors:** Isabelle Ottenvall Hammar, Synneve Dahlin-Ivanoff, Katarina Wilhelmson, Kajsa Eklund

**Affiliations:** Department of Clinical Neuroscience and Rehabilitation, Institute of Neuroscience and Physiology, The Sahlgrenska Academy, University of Gothenburg, Gothenburg, Sweden; Department of Physiotherapy and Occupational therapy, The Sahlgrenska University Hospital, Gothenburg, Sweden; Vårdalinstitutet, The Swedish Institute for Health Sciences, Universities of Gothenburg and Lund, Lund, Sweden; Department of Geriatrics, The Sahlgrenska University Hospital, Gothenburg, Sweden; Centre of Aging and Health-AGECAP, University of Gothenburg, Gothenburg, Sweden

**Keywords:** Aged 80 and over, Activities of daily living (ADL), Decision-making, Grounded theory, Sweden

## Abstract

**Background:**

Older persons’ right to exercise self-determination in daily life is supported by several laws. Research shows that older persons’ self-determination is not fully respected within the healthcare sector. In order to enable and enhance older persons’ self-determination, extensive knowledge of older persons’ self-determination is needed. The aim of this study was to explore experiences of self-determination when developing dependence in daily activities among community-dwelling persons 80 years and older.

**Methods:**

Qualitative interviews were performed in accordance with a grounded theory method, with 11 persons aged 84–95 years who were beginning to develop dependence in daily activities.

**Results:**

The data analysis revealed the core category, “Self-determination - shifting between self-governing and being governed”. The core category comprised three categories: “Struggling against the aging body”, “Decision-making is relational”, and “Guarding one’s own independence”. Self-determination in daily activities was related to a shifting, which was two-fold, and varied between self-governing and being governed by the aging body, or by others.

**Conclusions:**

The findings imply a need to adopt a person-centered approach where the older persons’ own preferences and needs are in focus, in order to enhance their possibilities to exercise self-determination.

## Background

In Sweden, as in other Western democracies, one has a legal right to exercise self-determination. This applies to younger as well as older persons, even though the persons are dependent on healthcare in several ways [1]. According to the Charter of the United Nations [2], and Swedish law [3, 4], healthcare should be based on respect for older persons’ self-determination. Persons have a greater influence on their own care through participation in decision-making regarding their care [5], which is crucial in a person-centered approach to care [6]. This approach prioritizes persons’ own views about their conditions and life situations [7]. In the healthcare sector, professionals are in general aware of the importance of older persons’ rights to self-determination and engagement in decision-making [8]. Although it is essential, a number of studies [9–12], indicate that healthcare professionals do not fully practice, and respect the older persons’ self-determination. This may be due to organizational barriers such as time pressure, and shortage of employees [10], or personal barriers such as lack of information [9, 10], and communication [9].

The concept of self-determination can be described as a person’s ability to think, choose, decide and act on his/her own [13]. There is a lack of a clear definition in the literature, and the concept is often used as a synonym for autonomy [14, 15]. According to a concept analysis [16], self-determination can be defined as a complex phenomenon, which comprises of having the ability, as well as enough knowledge, and control over the whole process, to act/decide on one’s own free will, and to have ethical/legal rights [16]. Exercising self-determination in daily life is not only a question of a legal right; it is also important for older persons’ health and well-being [8, 17–19]. A restriction in decision-making among dependent older persons increases the likelihood of experiencing a stressful life situation, a feeling of not being treated as a person, and not having basic needs fulfilled [20].

A reduced possibility to exercise self-determination can be associated with increased dependence [20, 21]. The ability to independently perform daily activities naturally deteriorates in advanced age [22]. Independency and the reserve capacity may also decline due to increased frailty [23, 24]. Frailty can be seen as a continuum from robustness, to pre-frail, and to the final full frailty phase [25]. The pre-frail phase is common among community-dwelling older persons [26]. Frailty has also been described as a dynamic concept, extending from not being frail, to being frail, and it is directly related to decreased ability to perform daily activities independently [27].

Research on self-determination from the perspective of older persons who are beginning to develop dependence in daily activities is limited. An earlier qualitative study [20], has demonstrated that older persons need to exercise self-determination, even when they are dependent on other persons, and that healthcare professionals need further knowledge to enable the dependent older persons to exercise self-determination. Frail older persons perceive self-determination as a phenomenon which changes throughout life. The ability to exercise self-determination is influenced by several conditions in the persons’ lives such as feelings of safety and security, having the opportunity to influence and being involved, and to be taken into account [28]. Following extensive literature review, no previous studies were found that focused on self-determination in the context of older persons during the development of dependence in daily activities. In order to enable healthcare professionals to work with person-centered care as their focus, while encouraging, and enhancing older persons’ self-determination, further research is needed. Therefore, the aim of this study was to explore experiences of self-determination when developing dependence in daily activities among community-dwelling persons 80 years and older.

## Methods

### Design

To explore older persons’ experiences of self-determination when developing dependence, a grounded theory method inspired by the developments of Charmaz [29] was applied. This version of grounded theory has its origin in the classic version developed by Glaser and Strauss [30], and in the later version by Strauss and Corbin [31]. Basic in grounded theory is the focus on actions and processes in relation to a specific phenomenon [29]. The method was chosen because it enables enhanced knowledge and a wider understanding of the complexity of the older persons’ own experiences of their self-determination during the process of developing dependence in daily activities.

### Participants

The sample was selected from a larger study entitled “Elderly persons in the risk zone” [32], addressing community-dwelling, independent persons, 80 years and older (n = 459), who were at risk of becoming frail, drawn from official registers in two urban districts in the city of Gothenburg, Sweden. In this study, a target selection was used when selecting persons, since the goal was to reach persons who during the course of the study had developed dependence in daily activities. To achieve a heterogeneous group, in accordance with grounded theory [29], persons of both genders, with varied ages, and marital status, while demonstrating dependence in only Instrumental Activities of Daily Living (I-ADL), or in both I-ADL, and Personal Activities of Daily Living (P-ADL) were selected. In addition, the persons were selected on the basis of having a variety of home help service, which included publically, and privately-financed help, as well as help by family and relatives.

The persons’ degree of dependence in daily activities, and their degree of frailty were initially assessed at the two year follow-up in the larger study [32]. Persons’ degree of dependence in P-ADL, and I-ADL, was assessed by using the ADL staircase [33, 34]. The ADL staircase [33, 34] is a cumulative scale where six P-ADL items (bathing, dressing, going to the toilet, transfer, feeding and continence), and four I-ADL items (cleaning, shopping, transportation, cooking) are included. Since continence is not considered an activity, nine out of the ten original items were assessed. Persons dependent of support in doing their laundry were also included. The degree of frailty was measured by using the following eight frailty indicators; weakness, fatigue, weight loss, physical activity, poor balance, slow gait speed, visual impairment and cognition, which in turn was categorized into non-frail (0 indicators), pre-frail (1–2 indicators), and frail (3 or more indicators) [32].

### Data collection and procedure

The selected persons received phone calls informing them of the purpose of the study, and about confidentiality. The persons who verbally accepted to participate received an information letter about the study, and signed an informed consent form before taking part. Face-to-face interviews were conducted by the first author in the participants’ homes, in accordance with an interview guide, between August 2012 and June 2013. The interview guide focused on how the participants experienced their self-determination when developing dependence in daily activities. The opening questions, concerning the meaning of self-determination, and the experiences of exercising self-determination when being dependent, were asked in each interview. As recommended in grounded theory [29], the questions that followed in the interview guide were developed throughout the study, as a result of the previous interview. The interviews were tape-recorded, and transcribed verbatim by the first author.

### Data analysis

The analysis started directly after the first interview, in accordance with grounded theory [29]. Detailed memos were written after each interview, and during analysis of the data. The principles of initial coding, focused coding, and constant comparison were used when analyzing the data [29]. The analysis started with the initial coding by using line-by-line coding. Free writing, a recommended version of prewriting [29], was also used in order to more thoroughly examine the data from the initial phase of the analysis. The free writing was then followed by the focused coding, with the purpose of synthesizing and explaining the initial codes [29]. To capture the essence of the participants’ experiences of their self-determination, all codes were finally compared and sorted into categories. The initial coding and focused coding, all memos, and the writing of the results, were performed by the first author in Swedish, and later translated into English, as the purpose was to stay true to the essence of the interviews. All four authors continually discussed the essence of the interviews in relation to the categories. The data was re-examined several times by moving back and forth between the analysis steps, while listening to the interviews, which finally resulted in a unanimous outcome.

In accordance with the grounded theory method [29], saturation was reached when no new codes were found during the analysis process, which in this study occurred after 11 interviews. The interviews lasted between 31 and 66 minutes. The participants were between 84–95 years, were beginning to develop frailty or were frail, and had varied home help service (Table 1). All participants were dependent in one or more daily activity with a timeframe from a few months, up to 18 months.

**Table 1:** **Demographics of the participants and dependence situation**

Participant	Gender	Age	Marital status	Dependent in ^1^	Help offered by	Degree of frailty ^2^
1	Male	87	Married	P-ADL/I-ADL	Relatives/privately	4 (Frail)
				(Cleaning, partly)	(Privately)	
				(Transportation)	(Relatives)	
				(Transfer, partly)	(Relatives)	
2	Male	90	Widower	I-ADL	Home care service	2 (Pre-frail)
				(Laundry)		
3	Female	89	Single	P-ADL/I-ADL	Home care service	4 (Frail)
				(Laundry)		
				(Cleaning)		
				(Transportation)		
				(Bathing)		
4	Female	95	Widow	I-ADL	Privately	4 (Frail)
				(Cleaning)		
5	Female	84	Married	I-ADL	Relatives/privately	1 (Pre-frail)
				(Cleaning)	(Privately)	
				(Shopping, partly)	(Relatives)	
6	Female	90	Single	I-ADL	Privately	3 (Frail)
				(Cleaning)		
7	Male	92	Widower	I-ADL	Relatives/home care service	1 (Pre-frail)
				(Cleaning)	(Home care service)	
				(Shopping, partly)	(Relatives)	
8	Male	91	Widower	I-ADL	Home care service	0 (non-frail)
				(Laundry)		
				(Cleaning)		
9	Female	89	Single	P-ADL/I-ADL	Home care service	2 (Pre-frail)
				(Laundry)		
				(Cleaning)		
				(Bathing, partly)		
10	Female	86	Widow	I-ADL	Privately	2 (Pre-frail)
				(Cleaning)		
11	Male	87	Married	I-ADL	Home care service	1 (Pre-frail)
				(Cleaning)		

^1^P-ADL = Personal Activities of Daily Living, I-ADL = Instrumental Activities of Daily Living.

^2^Frailty measured with the following frailty indicators: weakness, fatigue, weight loss, physical activity, poor balance, slow gait speed, visual impairment and cognition categorized into non-frail (0 indicators), pre-frail (1–2 indicators) and frail (3 or more indicators).

The Regional Ethical Review Board in Gothenburg (EPN Gothenburg Drn: 2012/401-12) approved the study. All participants signed an informed consent form, and received both written and verbal information about the study before they participated in interviews. The consent form contained the purpose of the study, and that all data of interest would be eventually published in a scientific paper. The permission to publish the data was also asked before the interviews started. The participants were advised that they could stop the interview if they felt exhausted.

## Results

### Self-determination – shifting between self-governing and being governed

**Figure 1:**
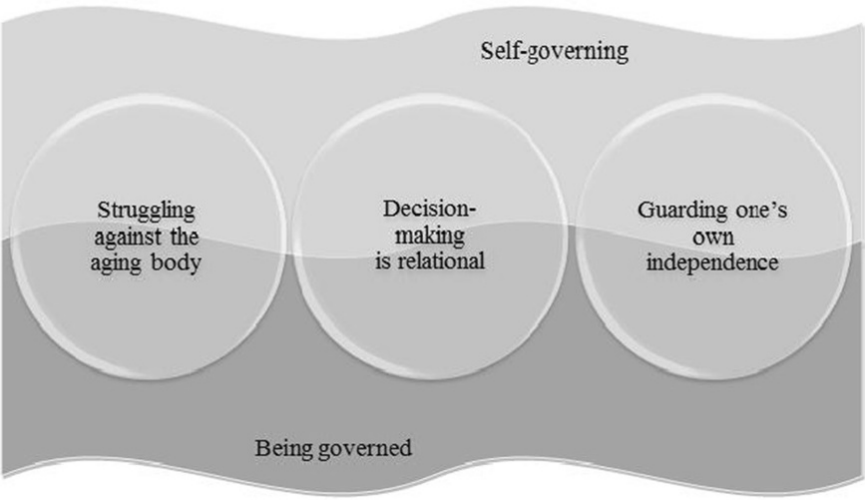
**A model illustrating self-determination as shifting between self-governing and being governed.**

The core category highlights the experiences of self-determination as a shifting process. The shifting, which is two-fold, moves from self-governing to being governed by the aging body, or by other persons. This is a non-linear process, which is constantly moving back and forth, depending on *which* activity is being performed, *who* is helping, and *how* extensive the help is. The specific type of activity being performed affects the possibility to self-govern and being governed. In addition, the person helping has an impact. Closer contacts versus superficial contacts generate a shifting that alternates between being the one who governs, to being governed. The extent of the help received also affects the governing in daily activities. A comprehensive need of help in daily activities may result in the greater likelihood of being governed by other persons. The shift of governing can be expressed in the three following categories: *Struggling against the aging body, Decision-making is relational,* and *Guarding one’s own independence* (Figure 1).

### Struggling against the aging body

Struggling against the aging body refers to the constant struggle against one’s own body, with the main purpose to continue exercising self-determination. By continuing the struggle, a feeling of being the one who governs arises, even though daily activities are successively becoming difficult to perform. Depending on the activity type, the person helping, and the extent of the help, the governing in daily activities shifts between self-governing, and being governed by the aging body. One woman describes the struggle that she is waging against her aging body:

“I can’t get into the bathtub, and I find it very hard to get up. I’ve tried letting out the water, and I’ve tried getting up when there was water left, but I can’t manage it. Eventually you get up, but it takes time. I think it’s a pity because it’s nice not to just shower. I think it’s so lovely to take a bubble bath” (Participant 10).

The shift of governing is linked to the awareness that the body gradually ages, a process that is unstoppable. Despite the struggle against the aging body, a number of activities must be sacrificed when it is no longer possible to govern over the aging body. Feelings of frustration may occur, when the shifting results in reduced possibilities to govern over the aging body. Even though there is a strong will to perform an activity, it is no longer possible to exercise self-determination over the aging body. The frustration occurs when the body, and not one’s own will, govern. This in turn reduces the possibilities to exercise self-determination over the aging body. A man, who is dependent while transferring, states the following strong frustration:

“Of course sometimes when I’m trying to get up, I say Damn! Damn (emphatically) that I can’t do anything myself. I must, I must. It happens, I must say that. Both when I’m lying [in bed] at night, I have to get up [and go to the toilet], with this prostate thing I have to get up and pee a little more often than most people, three times a night” (Participant 1).

### Decision-making is relational

Decision-making is relational, which means that the relationship between the person receiving, and the person offering help influences the possibility to exercising self-determination. The person who is helping has a direct impact on whether it is possible to continue to govern, or if the caregiver assumes the governing role. This fact generates a shift that is constantly moving back and forth. Depending on whether it is relatives, privately-financed help, or public home care service personnel who help with the specific daily activities, a shift of who is governing occurs. The shift also varies depending on which activity is being performed, and the extent of the help being received. Respect, consideration, personal chemistry, and attitude are essential features, which enable a continued governing in daily activities. Cooperation between the person receiving, and the person offering help, facilitates active participation in decision-making. When personal needs are fulfilled, the possibility to exercise self-determination will be enhanced. One woman, who has had both privately-financed help, and public home care services, highlights the importance of the relationship between the two parties:

“It’s like a way of co-operating, because sometimes she would say ‘What do you think? What do you think? Shouldn’t I take that thing there?’ (…). In other words, we had a co-operation (…). We simply respected each other. Respect is very important indeed” (Participant 6).

A mutual relationship between the parties involved can naturally lead to a transfer of the performance to another person. The transfer occurs when the specific help is satisfactory, and when decisions that have been planned, and carried out earlier are followed. By transferring the performance, but still continuing to participate, and receive questions regarding the daily activities that are performed by another person, the feeling is that it is still possible to self-govern. A man expresses how important it is to receive questions regarding what, and how an activity should be carried out:

“And you know she said to me right away that ‘Now you tell me what you want me to do’… I said the first time and since then it’s been like that the whole time” (Participant 7).

The possibility to exercise self-determination may be difficult when the relationship between the person receiving, and the person offering help, is not a good one. One woman, with earlier experiences of having public home care service with meals several times a day, characterizes the experiences of being governed by others:

“Then there was this person at breakfast, they could get here at nine o’clock. I said I wanted to have porridge and that I could do that myself. And I did that. Well, then she said (…) she was the one who should take care of that. She had gotten the idea that I wanted to eat a sandwich with cheese” (Participant 6).

### Guarding one’s own independence

Guarding one’s own independence means that the independence is guarded by only accepting help in the daily activities that are necessary to get help in. By constantly guarding, and not receiving more help than necessary, one gains a sense of control and feels that it is possible to govern. The degree of guarding varies depending on which activity is being performed, the person who is offering help, and the extent of the help. It is easier to let the guard down in I-ADL, than in P-ADL. As the degree of guarding differs, there is a constant shift between self-governing, and being governed by others. One way to guard one’s own independence is by consulting trustworthy persons like family and friends. Increased knowledge generates a sense of security, which makes it easier to govern in daily activities. The advice may consist of basic everyday activities, but can also be applied to more advanced activities, such as technology and electrics. A man who often asks his relatives for advice, states the following:

“If there are specific things I need to buy, I seek advice. I have a curtain that I took down in the bedroom, and I’m going to put up a valance there. In that case I asked one of my girls to give me advice about getting a new valance” (Participant 8).

An increased dependence in daily activities may result in an extended help from relatives, friends and neighbors. One has to constantly be on guard in order to not receive more help than necessary. The guarding is associated with the desire not be intruding, and not to be dependent on relatives, friends and neighbors. One woman expresses the desire not to receive more help than needed:

“… But of course you don’t want to overdo it and bother too much. It’s never been so that they [the neighbors] have ever said no, we don’t want to, and they’ve always been so kind. But you have to understand that you can’t depend on their help, because then you intrude on their freedom. They shouldn’t feel obligated” (Participant 3).

## Discussion

This study revealed that self-determination, during the development of dependence, was experienced as an ongoing and shifting process. The shifting constantly moved back and forth, between self-governing, and being governed by either the aging body, or by others, depending on the specific activity, the persons involved, and the extent of help.

The aging body affected the possibility to exercise self-determination in daily activities, which resulted in a struggle against one’s own body. Along with that struggle, the participants guarded their independence for maintaining their self-determination in daily activities. According to a study by Haak et al. [35], older persons struggled to be independent because it was important for their self-confidence, regardless of degree of functional decline. Furthermore, the findings emphasize the importance of the relationship between the person receiving help, and the person offering help. An earlier study [36], has shown that unbalanced relationships may result in experiences of powerlessness, and loss of self-determination for the persons receiving help [36].

The present study showed that self-determination is a rather complex, and dynamic phenomenon, which may shift from time to time, and differ between different activities. The community-dwelling, dependent older persons were not able to self-govern their daily activities for several reasons. On occasion, healthcare professionals governed the participants, instead of letting the participants self-govern. With this in mind, more attention must be put on older persons’ self-determination. According to Cardol [37], decisional autonomy, i.e. self-determination, should have a central place in rehabilitation. By striving for the highest level of self-determination, the possibility for the older persons to participate in daily life could be promoted [37]. Moreover, a person’s uniqueness must be respected and understood, which could be accomplished by adopting a person-centered approach [6]. This approach is holistic and respectful, offering options through a therapeutic relationship, where persons receiving care are empowered to be involved in decisions-making. Empowerment, a central attribute in person-centered approach to care [6], promotes self-confidence, which in turn gives enriched possibilities to experience self-determination [5]. Following this approach, professionals should offer practical expertise and personal support, and also enable persons to follow their own choices [38].

In this study, the participants experienced a need to govern their independence in relation to other persons. In this context, the central focus should be on a person’s capabilities; on what a person is able to do, and able to be [39]. With a focus on persons’ capabilities, everyone should be viewed as unique persons, and not as a group [39], which is in line with the person-centered approach to care [5–7, 38]. The capability approach was developed by Sen [40], and further developed by Nussbaum [39]. Fundamental for this approach is the focus on life, as comprising a set of interrelated functionings, which are based on beings, and doings [41], like happiness, good health, and participation in community-life [41, 42]. Healthcare professionals need to constantly protect, and support the dependent, older persons’ capabilities to continue to perform daily activities. It is also important to constantly enhance self-determined capabilities in daily life, irrespectively of being independent or not. With respect to this, the variety of functions that are of importance for human life should be taken into account [39].

The primary focus in this study was on experiences of self-determination among persons who were in the process of developing dependence in daily activities. Therefore, a grounded theory method was used since it is a suitable method when processes and actions are in focus [29]. Furthermore, qualitative studies depend on the researcher. Reflexive strategies could be a way to handle the authors’ preconceptions [29]. The first author’s personal preconceptions, working in research and clinical settings with older persons, might have influenced the interviews and analysis.

A grounded theory method inspired by the developments of Charmaz [29] was used in this study. According to Charmaz [29], there are four central criteria for quality in a grounded theory study; credibility, originality, resonance and usefulness. The credibility can be based on the richness of the data [29]. Eleven persons with diverse dependence in daily activities were included, which contributed to a wide range of experiences. Also, the strong links between the data and the analysis are ways of attaining credibility [29]. All authors listened to the interviews and participated in the discussions. In order to stay close to the participants’ own words, both the analysis, and the writing of the results were performed in the authors’ native language. The criteria of originality addresses whether the study offers new insight [29]. The core essence indicated that the community-dwelling older persons experienced their self-determination in daily life as a constant ongoing shifting process. The shifting process was directly related to three central parts; the specific activity, the persons involved, and the extent of help. The manner in which these three parts interacted, and the fact that they had a direct impact on the shifting process, has not been described earlier. This new insight confirms that this study meets the criteria of originality.

Charmaz [29], means that if the fullness of the studied phenomenon is captured, the criteria of resonance is fulfilled in a study. Both the heterogeneity of the sample, and the complexity of the findings, are valid when analyzing the resonance of this study. By using a target selection, and recruiting a heterogeneous group in terms of age, gender, marital status and degree of dependence, a diversity of experiences of self-determination were described. Moreover, the findings highlight the complexity of the studied phenomenon. It shows that self-determination is a non-static concept that may vary widely, depending on many contributing factors. Based on this, the resonance of this study should be achieved.

Finally, the participants were selected from a larger study [32] with persons from two urban districts in Gothenburg. While analyzing the usefulness of this study, one must remain mindful that the two urban districts, from which the sample was taken, represent persons born in Sweden with a slightly higher income and education level, and less sickness. Moreover, the meaning of self-determination varies between different countries. In the Western society, there is comparatively more individual decision-making, whereas in the Eastern society the trend is a family-determined principle [43]. The findings in this study only represent experiences among older persons from one narrow context, and for that reason further studies are needed in which other cultures and contexts are included. The contribution of the findings must be addressed when analyzing the usefulness [29].

The findings have not generated a model, or a theory construction. However, an analytic framework based on the participants’ own experience is put forth. Charmaz [29], argues that the creation of a model, or a theory is not the main focus in a grounded theory study. Instead, focus should be on the exploration of the phenomenon [29].

## Conclusions

For community-dwelling older persons, experiences of self-determination when developing dependence were related to a shift between self-governing, and being governed by the aging body, or by others. Depending on the specific activity, the person offering help, and the extent of help, self-determination was attainable to a greater or lesser extent. The relationship between the persons involved had a direct impact on whether it was possible to continue to exercise self-determination in daily life, or not. Based on this, healthcare professionals and healthcare providers should work more actively to enable, and encourage dependent older persons to exercise self-determination. By adopting a person-centered approach, with a focus on a person’s capabilities, the older persons could continue to exercise self-determination, even though they demonstrate dependence in daily activities.
